# HDAC1-3 inhibition increases SARS-CoV-2 replication and productive infection in lung mesothelial and epithelial cells

**DOI:** 10.3389/fcimb.2023.1257683

**Published:** 2023-12-13

**Authors:** Flavia Trionfetti, Tonino Alonzi, Giulio Bontempi, Michela Terri, Cecilia Battistelli, Claudia Montaldo, Federica Repele, Dante Rotili, Sergio Valente, Clemens Zwergel, Giulia Matusali, Fabrizio Maggi, Delia Goletti, Marco Tripodi, Antonello Mai, Raffaele Strippoli

**Affiliations:** ^1^ Department of Molecular Medicine, Sapienza University of Rome, Rome, Italy; ^2^ Gene Expression Laboratory, National Institute for Infectious Diseases, Lazzaro Spallanzani IRCCS, Rome, Italy; ^3^ Translational Research Unit, National Institute for Infectious Diseases “Lazzaro Spallanzani”-IRCCS, Rome, Italy; ^4^ Department of Drug Chemistry and Technologies, Sapienza University of Rome, Rome, Italy; ^5^ Laboratory of Virology, National Institute for Infectious Diseases, Lazzaro Spallanzani IRCCS, Rome, Italy; ^6^ Pasteur Institute, Cenci-Bolognetti Foundation, Sapienza University of Rome, Rome, Italy

**Keywords:** HDAC (histone deacetylase), SARS-CoV-2, ACE2 regulation, TMPRSS2 expression, mesothelial cells, pleura, viral infection and replication, epidrugs

## Abstract

**Background:**

Despite the significant progress achieved in understanding the pathology and clinical management of SARS-CoV-2 infection, still pathogenic and clinical issues need to be clarified. Treatment with modulators of epigenetic targets, i.e., epidrugs, is a current therapeutic option in several cancers and could represent an approach in the therapy of viral diseases.

**Results:**

Aim of this study was the analysis of the role of histone deacetylase (HDAC) inhibition in the modulation of SARS-CoV-2 infection of mesothelial cells (MCs).

MeT5A cells, a pleura MC line, were pre-treated with different specific class I and IIb HDAC inhibitors. Unexpectedly, treatment with HDAC1-3 inhibitors significantly increased ACE2/TMPRSS2 expression, suggesting a role in favoring SARS-CoV-2 infection. We focused our analysis on the most potent ACE2/TMPRSS2 inducer among the inhibitors analysed, MS-275, a HDAC1-3 inhibitor. ACE2/TMPRSS2 expression was validated by Western Blot (WB) and immunofluorescence. The involvement of HDAC inhibition in receptor induction was confirmed by HDAC1/HDAC2 silencing. In accordance to the ACE2/TMPRSS2 expression data, MS-275 increased SARS-CoV-2 replication and virus propagation in Vero E6 cells.

Notably, MS-275 was able to increase ACE2/TMPRSS2 expression and SARS-CoV-2 production, although to a lesser extent, also in the lung adenocarcinoma cell line Calu-3 cells.

Mechanistically, treatment with MS-275 increased H3 and H4 histone acetylation at ACE2/TMPRSS2 promoters, increasing their transcription.

**Conclusion:**

This study highlights a previously unrecognized effect of HDAC1-3 inhibition in increasing SARS-CoV-2 cell entry, replication and productive infection correlating with increased expression of ACE2 and TMPRSS2. These data, while adding basic insight into COVID-19 pathogenesis, warn for the use of HDAC inhibitors in SARS-CoV-2 patients.

## Introduction

At the end of 2019, a novel coronavirus subsequently known as severe acute respiratory syndrome (SARS)-CoV-2 was first identified in Wuhan, China, causing the outbreak of an unusual pneumonia rapidly evolving into a pandemic spreading around the world with an enormous death toll (Andersen et al., 2020; Wu et al., 2020). SARS-CoV-2 clinical manifestations range from asymptomatic infection to mild disease with symptoms to severe pneumonia, acute respiratory distress syndrome (ARDS), and multiple organ failure with different therapeutic options (Cantini et al., 2020; Hu et al., 2021; Yang et al., 2021; Ferraccioli et al., 2022).

The first encounter between the host and SARS-CoV-2 occurs in epithelial cells of the upper respiratory tract (nasal passages and throat) and especially lungs (bronchi and alveoli). In the lungs, the alveolar type I and type II cells (AT1 and AT2, respectively) play a significant role in SARS-CoV-2 infection. Moreover, alveolar macrophages are determinant in mediating the subsequent amplification of the inflammatory and immune response (Grant et al., 2021; Najafi Fard et al., 2021; Aiello et al., 2023). Besides the best-studied tissues and organs, SARS-CoV-2 may spread to other organs that may act as a virus reservoir, whose biological meaning is still uncertain (Cheung et al., 2022).

Viral entry in host cells is mediated by the engagement of angiotensin-converting enzyme 2 (ACE2), the main receptor implicated in SARS-CoV-2 recognition (Hoffmann et al., 2020). ACE2 is expressed by cells in lungs but also in endothelium, in kidney and heart cells, whose infection by SARS-CoV-2 may mediate the characteristic multi-organ pathology (Li MY. et al., 2020; Zou et al., 2020).

SARS-CoV-2 uptake is also mediated by transmembrane protease serine (TMPRSS)2, whose activity is necessary to prime Spike protein to allow virus-plasma membrane fusion (Hoffmann et al., 2020; Najafi Fard et al., 2021). Besides ACE2 and TMPRSS2, other receptors, including ADAM17 and NRP1, have been demonstrated to potentiate SARS-CoV-2 entry and infectivity (Cantuti-Castelvetri et al., 2020; Hoffmann et al., 2020). However, ACE2-independent SARS-CoV-2 has been shown to play a role in the infection of T lymphocytes (Shen et al., 2022).

It was previously demonstrated that pleura mesothelial cells (MCs) express ACE2 and TMPRSS2 receptors. In these cells, SARS-CoV-2 infection resulted in the production of a broad repertoire of interferons, pro- and anti-inflammatory cytokines, and metalloproteases (MMPs) actively participating in the immune response to infections. MC infection by SARS-CoV-2 may play a role in extracellular matrix (ECM) remodeling and immunomodulation (Matusali et al., 2021; Terri et al., 2021; Trionfetti et al., 2023).

Chemical covalent modifications of histone proteins drive the shift from heterochromatin to euchromatin and vice versa, regulating by this way gene expression in eukaryotic cells (Mai, 2010; Ferguson et al., 2011; Zwergel et al., 2015). Increased lysine acetylation at histone level is generally found in transcriptionally active genes, whereas hypoacetylation is associated with transcriptionally silent genome regions.

The balance between acetylation and deacetylation of histones as well as non-histone proteins is regulated by two key enzyme families, histone acetyltransferases (HATs) and histone deacetylases (HDACs), which are components of multiprotein complexes containing other proteins known to exert their role in transcriptional activation/repression. To date, eighteen distinct human HDACs have been reported, grouped into four classes (I-IV) depending on their primary homology to *Saccharomyces cerevisiae* HDACs (RPD3, HDA1, and SIR2) (Lakshmaiah et al., 2014; Di Bello et al., 2022).

Pharmacological inhibition of HDAC activity is a current therapeutic option in cancer management and could represent a promising approach in viral diseases (Lakshmaiah et al., 2014; Panella et al., 2016).

Besides pan-HDAC inhibitors such as trichostatin A and vorinostat, small molecules have been designed to selectively inhibit the activity of specific HDAC classes/isoforms (Ho et al., 2020). Epigenetic inhibitors have been demonstrated to impact SARS-CoV-2 biology and have been suggested as a potential therapeutic strategy (Liu et al., 2020; Ripamonti et al., 2022). Also, the repurposing of currently used HDAC inhibitors has been suggested (Pitt et al., 2021). However, there is a lack of mechanistic details to clarify the effect of HDAC inhibition in SARS-CoV-2 virus-host interactions.

This study aims to analyze the role of class I and class IIb HDAC inhibitors in SARS-CoV-2 infection, focusing on their role in ACE2 and TMPRSS2 expression in a MC line. MS-275, a pharmacological HDAC1-3 inhibitor, was found to markedly increase SARS-CoV-2 replication and productive infection through the induction of ACE2 and TMPRSS2 expression. We believe these discoveries are relevant from both a basic and translational point of view.

## Materials and methods

### Cell culture and treatment

The human mesothelial cell line MeT5A (ATCC, Rockville, MD) was cultured in Earle’s M199 supplemented with 10% FBS (Gibco-Life Technologies), 2 mM L-glutamine (EuroClone), 100 U/ml penicillin, 100 µg/ml streptomycin (Gibco-Life Technologies). This cell line was isolated from pleural fluids obtained from a non-cancerous individual. The human adenocarcinoma cell line Calu-3 was grown in RPMI 1640 (Euroclone, Milan, Italy; cat. n°ECB9006L) supplemented with 10% FBS, 2 mM L-glutamine, (EuroClone), 100 U/ml penicillin, 100 µg/ml streptomycin (Gibco-Life Technologies).

Cell lines were grown at 37°C, in a humidified atmosphere with 5% CO_2_. MeT5A or Calu-3 cells cells were treated with DMSO or MS-275 in a dose-dependent experimental model ranging between 1 to 0.06 μM or 5 to 0.06 μM, respectively. MeT5A cells were treated with the epigenetics drugs MS-275, MC-2468, MC-4448, Tubastatin A, PCI34051, MC-2500 at 0.25μM and MGCD0103 at 0.125μM. Pharmacological treatment was repeated every 48 hours during the experimental procedure.

### Antibodies and chemicals

The primary antibodies for western blotting (WB) experiments: mAbs anti-GAPDH (sc-32233) and -Histone H4, were from Santa Cruz Biotechnology (Dallas, TX). pAbs anti–ACE2 (AB_2792286) was from Invitrogen (Waltham, MA). pAbs anti-TMPRSS2 (ab109131) was from Abcam (Cambridge, UK). Ab anti-acetyl-H3 (06599) was from Millipore (Merk, Kenilworth, NJ). Ab anti-acetyl-H4 (06866) was from Millipore (Merk, Kenilworth, NJ); control rabbit IgG (NB810-56910) was from Novus Bio, Minneapolis, MN.

Antibodies for Immunofluorescence: anti-ACE2 (Thermo Fisher Scientific, Waltham, MA, USA, AB_2792286). Cy3-conjugated anti-rat secondary antibodies (Jackson ImmunoResearch, 112-165-003). DRAQ5 staining solution (#130-117-343) was from Milteny. MS-275, MGCD0103, MC-2468, MC-4448, Tubastatin A, PCI34051, MC-2500 were from Prof. Mai laboratory. The VSV-spike-GFP construct was a kind gift from Sean Whelan, Washington University St. Louis, USA.

### Viral infection

Subconfluent MeT5A (2.0×10^5^ cells/well; 24 wells) or Calu-3 (3.75x10^5^ cells/well; 24 wells) were pretreated with different doses of either MS-275 or DMSO as control, 24 hours (h) before infection.

Cells were then incubated with SARS-CoV-2 (isolate SARS-CoV-2/Human/ITA/PAVIA1073 4/2020, clade G, D614G (S) obtained from Dr. Fausto Baldanti, Policlinico San Matteo, Pavia, Italy) in serum-free Eagle’s Minimum Essential Medium at a multiplicity of infection (MOI) of 1 for MeT5A cells and MOI 0.001 for Calu-3 cells for 1 h at 37°C, 5% CO_2_. Then, cells were washed with 1X PBS to remove viral inoculum, and complete culture medium supplemented by DMSO or MS-275 was added. Fresh DMSO or MS-275 was added 48 h post-infection. Culture supernatants and cell lysates were collected at 72 h post-infection (p.i.) (MeT5A cells) or 24 h and 48 h p.i. (Calu-3 cells).

### Viral titration

To estimate the production of infectious SARS-CoV-2, serial dilutions of either MeT5A or Calu-3 cell culture supernatants, treated either with DMSO or MS-275, were measured by back-titrating assay in Vero E6 cells, as we recently described (Alonzi et al., 2021; Alonzi et al., 2022). Readout of the virus back-titration was based on detection of cytopathic effect (CPE), and infectious titer was expressed as 50% tissue-culture infective dose (TCID50) values, calculated according to the Reed–Muench method.

### Viral entry

MeT5A cells were pretreated with MS-275 or DMSO as control for 48 hours before infection. Cells were then incubated for 1 hour with VSV-spike-GFP construct at MOI=1 to evaluate Spike-dependent viral entry. Then, cells were washed three times with 1X PBS to remove the viral inoculum and fixed with 4% paraformaldehyde (Merck Life Science, Milan, Italy) in 1X PBS to perform an immunofluorescence assay.

### siRNA-mediated knockdown

siGENOME SMARTpool siRNAs HDAC1(3065), siHDAC2 (3066) and siRNA control were purchased from Dharmacon. 200×10^3^ MeT5A cells were seeded on 12-well plates 24 h prior to transfection. Cells were transfected with 25 pmol of siRNAs, and 3.5 μl Lipofectamine^®^ RNAiMAX Reagent (Thermo Fisher Scientific) were diluted in two different tubes with 200 μl Opti-MEM (Gibco-Life Technologies). The two solutions were mixed gently and were incubated for 20 minutes at room temperature. Transfection solutions were added to cells with 0.6 ml of supplemented medium. Efficiency transfection was evaluated using RT-PCR.

### Reverse-transcriptase polymerase chain reaction

Cellular RNA was extracted from cell cultures using TRIzol reagent (Life Technologies, Carlsbad, CA), according to the manufacturer’s instructions. cDNA synthesis was generated using a reverse transcription kit (A3500) from Promega (Madison, WI), according to the manufacturer’s recommendations. cDNAs were amplified by qPCR reaction using Maxima SYBR Green/ROX qPCR Master Mix (K0253) from Thermo Fisher Scientific (Waltham, MA). qPCR reactions were performed with the Rotor-Gene 6000 thermocycler (Corbett Research, Cambridge, United Kingdom). The primer sequences used in this study are shown in **Table 1**. Relative amounts obtained with 2 (−ΔCt) method were normalized with respect to the housekeeping gene L34. Statistical significance was determined with a t-test with Prism version 8.0. Differences were considered significant at p < 0.05.

**Table 1:** List of primers used in the study.

Target gene	Forward Sequence	Reverse Sequence
ACE2	GGGATCAGAGATCGGAAGAAGAAA	AGGAGGTCTGAACATCATCAGTG
HDAC1	CATCGCTGTGAATTGGGCTG	CCCTCTGGTGATACTTTAGCAGT
HDAC2	GAATCCGCATGACCCATAAC	TTCTTCGGCAGTGGCTTTAT
TMPRSS2	AATCGGTGTGTTCGCCTCTAC	CGTAGTTCTCGTTCCAGTCGT
L34	GTCCCGAACCCCTGGTAATAGA	GGCCCTGCTGACATGTTTCTT
ACE2 promoter	GTCCCCTGTGAGCCAAGAT	AACCCAAGTTCAAAGGCTGA
TMPRSS2 promoter	GCATCTCAGCGAGTTTCCAG	GTGCGCCTTTTCTCTTTGGG
RPL30 promoter	GCAGGAAGATGGTGGCCGCAA	AGTCTGCTTGTACCCCAGGACGT

### MTT assay

To evaluate cell viability upon MS-275 treatment Met5A cells (5.000 cells/well) and Calu-3 cells (20.000 cells/well) were stimulated with MS-275 for 48 hours and then tested with CyQUANT™ MTT Cell Viability Assay (V13154) from Invitrogen (Waltham, MA, USA) following manufacturer instruction. Absorbance were measured at 480 nm with Microplate Reader from Biorad (Hercules, CA, USA).

### Western blotting

MeT5A cells were lysed in CelLytic™ MT Cell Lysis Reagent supplemented with 1 mM PMSF; 1 μg/ml each of aprotinin, leupeptin, and pepstatin; and 25 mM NaF (all from Merck Life Science). For histone extraction, MeT5A cells were lysed in Triton Extraction Buffer (TEB: PBS containing 0.5% Triton 100x, 2mM PMSF and 0.2% NaN_3_). Pellet was suspended in 0.2N HCl. Equal amounts of protein were resolved by SDS-PAGE. Proteins were transferred to nitrocellulose membranes (Amersham Life Sciences, Little Chalfont, United Kingdom) and probed with primary antibodies using standard procedures. Peroxidase–conjugated secondary antibodies anti-rabbit (711-036-152) and anti-mouse (715-036-150) were from Jackson Immuno Research Laboratories (West Grove, PA, United States). Nitrocellulose-bound antibodies were detected by enhanced chemiluminescence (ECL) Immobilon Classico WBLUC0500 and Immobilon Crescendo Western HRP substrate WBLUR0500 from Millipore (Burlington, MA, United States).

### Immunofluorescence and confocal microscopy

After pharmacological treatments or infections, cells were washed in cold PBS, fixed with 4% paraformaldehyde (Merck Life Science) in 1X PBS, and permeabilized with 0.2% Triton X-100 (Merck Life Science) in 1X PBS. Coverslips were mounted in Prolong Gold antifade (Life Technologies) and examined under confocal microscopes (Leica TCS SP2, Wetzlar, Germany and Celldiscoverer 7, Carl Zeiss AG, Oberkochen, Baden-Württemberg, Germany). Digital images were acquired with the Leica software, and the image adjustments and merging were performed using the appropriate tools of ImageJ software. A minimum of 4 fields per sample (at least 150 total cells per total) from two independent experiments was analyzed.

### ChIP assay

Chromatin crosslinking and sonication were performed as in (Battistelli et al., 2017). After determining the DNA concentration, 50 μg of chromatin were used for each sample. It was diluted 10 folds with dilution buffer (TE 1x, sodium dodecyl sulfate (SDS) 0.5%, and protease and phosphatase inhibitors) and was incubated overnight with 5 μg of acetyl-H3, acetyl-H4 or control rabbit IgG and 20 μl magnetic beads. Immuno-precipitated samples were put on a magnetic rack, and Input sample was recovered by the supernatant of the IgG control. The samples were washed with four successive buffers: I) Low salt Buffer, II) High salt Buffer, III) LiCl Buffer, IV) TE Buffer. During the washes, the samples were in rotation, and at the end of each wash, magnetic beads were recovered. Then, the samples were dissolved in 300 μl of elution buffer (1% SDS, 0.1M NaHCO_3_) and 10 mg of RNase A; 0.5% SDS and RNase were added to the Input sample for 10 min at RT. 30 μl of Proteinase K (10 mg/ml) were added to all samples (included input). Elution, proteinase K and reversal crosslink steps were performed at 62°C for 5 hours while vortexing. DNA extraction was performed like conventional ChIP, see above. Finally, DNA was resuspended in 50 μl of nuclease-free water.

## Results

### Treatment with Class I and IIb HDAC inhibitors modulates the expression of ACE2 and TMPRSS2 SARS-CoV-2 specific receptors

**Figure 1:**
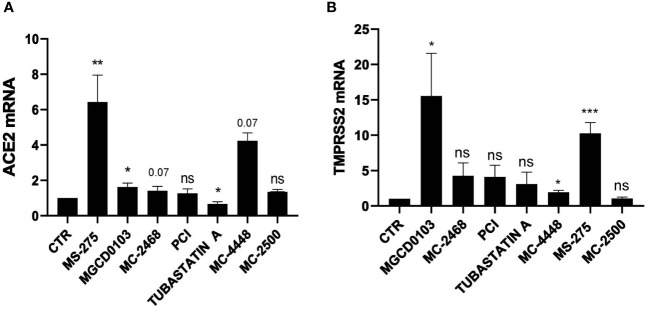
Treatment with class I and IIb HDAC inhibitors modulated the expression of ACE2 and TMPRSS2 mRNA. MeT5A cells were treated for 48 hours with the following epigenetic inhibitors: MGCD0103 (125 nM), MC2468 (250 nM), PCI-34051 (250 nM), Tubastatin A (250 nM), MC4448 (250 nM), MS-275 (250 nM), MC2500 (250 nM). Quantitative RT-PCR expression analysis of *ACE2* **(A)** and *TMPRSS2* **(B)** was performed from total RNA of treated MeT5A compared to CTR (DMSO, 250 nM). L34 mRNA levels were used for normalization. Bars represent the mean ± SEM of triplicate determinations from six independent experiments. *P* was calculated with respect to CTR. Differences were considered significant at *p* < 0.05. *P< 0.05; **P*P*< 0.01; ***P*P*< 0.001; ns, not significant.

To analyze the impact of HDAC in SARS-CoV-2 infection, we first analyzed the expression of the main receptors ACE2 (**Figure 1A**) and TMPRSS2 (**Figure 1B**) upon treatment of MeT5A cells with seven specific pharmacological inhibitors with different class/isoform selectivity. Treatment with the HDAC1/2 inhibitor Mocetinostat (MGCD0103) (Fournel et al., 2008; Khan et al., 2008), the HDAC1-3 inhibitor Entinostat (MS-275) (Saito et al., 1999; Suzuki et al., 1999; Rossi et al., 2018), the HDAC3 inhibitor MC4448, the class I/IIb HDAC inhibitor MC2468 (Zwergel et al., 2021), the HDAC6 inhibitor Tubastatin A (Butler et al., 2010), and the HDAC8 inhibitor PCI-34051 (PCI) (Balasubramanian et al., 2008) differently modulated the expression of these receptors. Interestingly, MGCD0103 and MS-275 induced a significant increase of both ACE2 and TMPRSS2, whereas treatment with compound MC2500 (Suzuki et al., 1999; Rossi et al., 2018), an inactive *meta* isomer of MS-275, did not modify the expression of these receptors. Of note, the HDAC3-selective inhibitor MC4448 induced the expression of both ACE2 and TMPRSS2. On the other hand, treatment with the HDAC6-selective inhibitor Tubastatin A significantly downregulated ACE2, while not affecting TMPRSS2 expression. Treatment with the HDAC8-selective inhibitor PCI-34051, as well as the class I/IIb HDAC inhibitor MC2468, was not effective. These results highlight the different activity of isoform-specific HDAC inhibitors regarding ACE2 and TMPRSS2 expression, emphasizing the role of HDAC1-3 inhibition in this context.

### Treatment with either MS-275 or genetic silencing of HDAC1 and HDAC2 enhances the expression of ACE2 and TMPRSS2

**Figure 2:**
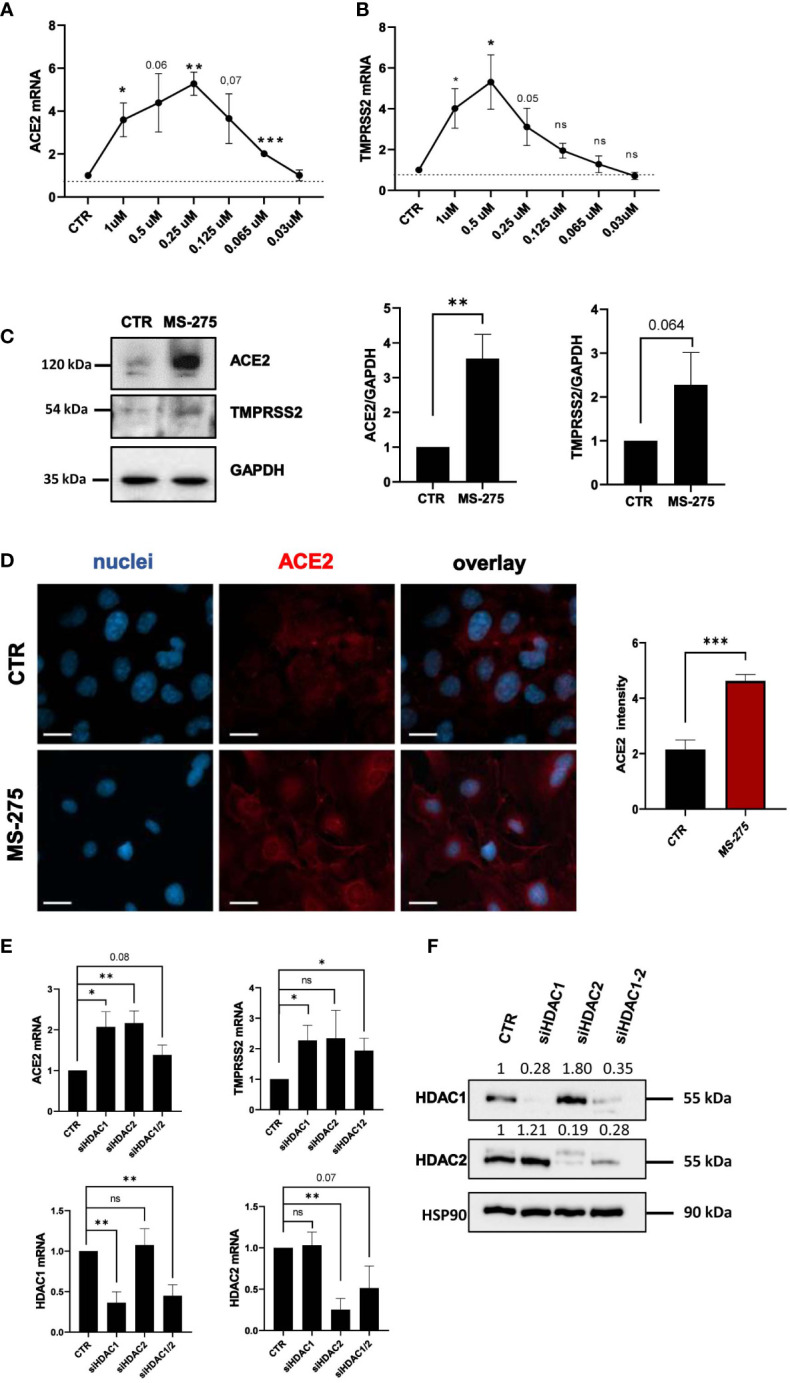
Treatment with MS-275 and genetic silencing of HDAC1 and HDAC2 enhances the expression of ACE2 and TMPRSS2. **(A, B)** MeT5A cells were treated with DMSO or MS-275 for 48 hours at the following doses: 1, 0.5, 0.25, 0.125, 0.065, and 0.03 μM. Quantitative RT-PCR expression analysis of *ACE2* and *TMPRSS2* was performed from total RNA of MS-275-treated MeT5A cells compared to DMSO-treated samples (CTR). L34 mRNA levels were used for normalization. Bars represent the mean ± SEM of triplicate determinations from three independent experiments. *P* was calculated with respect to CTR. Differences were considered significant at *p* < 0.05. *P< 0.05; **P< 0.01; ***P< 0.001; ns, not significant. **(C)** Left: Western blot showing the expression of ACE2 and TMPRSS2 from total cellular extracts of MeT5A cells treated for 48 hours with 0,25 μM DMSO or 0.25 μM MS-275. GAPDH was detected as a loading control. Right: quantification of ACE2 and TMPRSS2 protein expression. Bars represent the mean ± SEM of triplicate determinations in four independent experiments. *P* was calculated with respect to CTR samples. **(D)** Immunofluorescence of MeT5A cells treated with 0.25 μM MS-275 for 48 hours compared to CTR. Left: Fixed cells were stained with antibodies against ACE2. Nuclei were stained with DRAQ5. A minimum of 150 cells per sample from two independent experiments were analyzed. Scale bar: 25 μm. Right: ACE2 fluorescence signal quantification in MS-275 treated cells compared to CTR. Bars represent the mean ± SEM of triplicate determinations. *P* was calculated with respect to CTR-infected samples. Differences were considered significant at *p* < 0.05. *P< 0.05; **P< 0.01; ***P< 0.001; ns, not significant. **(E)** Cells were treated with gene silencing SMARTpool to silence HDAC1 and/or HDAC2 gene expression. Quantitative RT-PCR expression analysis of *ACE2*, *TMPRSS2, HDAC1*, and *HDAC2* from total RNA of treated MeT5A cells compared to control treatment (CTR). L34 mRNA levels were used for normalization. Bars represent the mean ± SEM of triplicate determinations from six independent experiments. *P* was calculated with respect to CTR samples. Differences were considered significant at *p* < 0.05. *P< 0.05; **P< 0.01; ***P< 0.001; ns, not significant. **(F)** Western blot showing the expression of HDAC1 and HDAC2 from total cellular extracts of genetically silenced MeT5A cells. HSP90 was detected as a loading control.

We then focused on MS-275, which was the most potent HDAC inhibitor in ACE2 modulation. Dose-response experiments confirmed the induction of ACE2 and TMPRSS2 gene expression, with a peak of induction at 0.25 µM and 0.5 µM, respectively (**Figures 2A, B**). The decreased expression of ACE2 and TMPRSS2 observed at higher doses of MS-275, are likely due to a significant reduced cellular viability (**Supplementary Figure 1A**). ACE2 and TMPRSS2 expression was confirmed at protein level by Western Blot analysis (**Figure 2C**) and by confocal microscopy (**Figure 2D**). The specific genetic silencing of HDAC1 and HDAC2 isoforms confirmed that both HDAC1 and HDAC2 are involved in the expression of ACE2 and TMPRSS2 (**Figures 2E, F**).

### Treatment with MS-275 potentiates SARS-CoV-2 cell entry, replication and virion production

**Figure 3:**
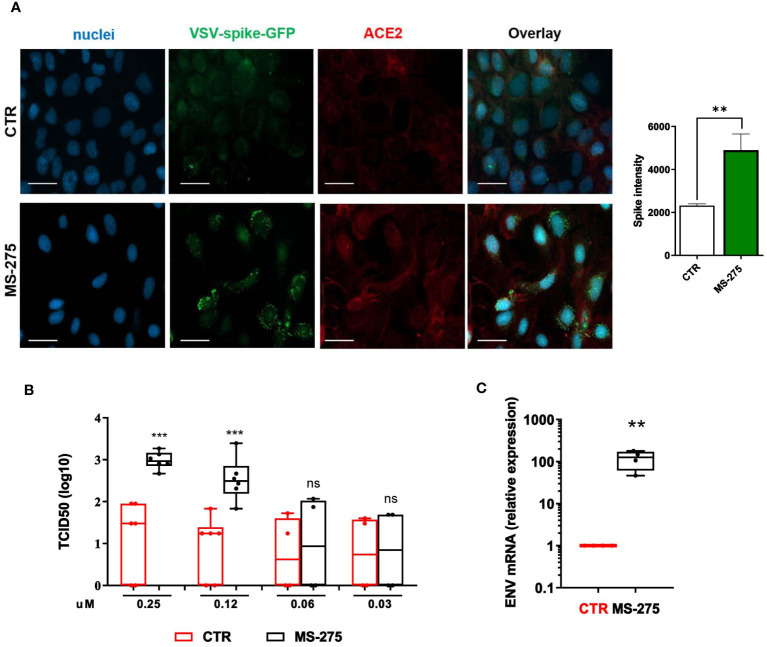
Treatment with MS-275 potentiates SARS-CoV-2 replication and productive infection in MeT5A cells. **(A)** Left: Immunofluorescence of MeT5A cells treated with 0.25 μM MS-275/DMSO for 48 h and incubated with VSV-Spike-GFP for 1 (h) Fixed cells were stained with antibodies against ACE2. Nuclei were stained with DRAQ5. A minimum of 150 cells per sample from two independent experiments were analyzed. Scale bar: 50 μm. Right: Spike-GFP fluorescence signal quantification in MS-275 treated cells compared to CTR. Bars represent the mean ± SEM of triplicate determinations. *P* was calculated with respect to CTR-infected samples. Differences were considered significant at *p* < 0.05. **P< 0.01; ***P< 0.001; ns, not significant. **(B)** MeT5A cells were pre-treated with DMSO or MS-275 for 24 hours at the following doses: 0.25, 0.125, 0.06, and 0.03 μM. Then, cells were infected with SARS-CoV-2 for 72 hours (MOI=1). TCID50 (Median Tissue Culture Infectious Dose) assay was performed by adding serial dilutions of MeT5A (MS-275- or DMSO-treated) cell culture supernatants to sub-confluent VeroE6 cells seeded in 96-well plates. Six independent experiments were performed. **(C)** Quantification of SARS-CoV-2 ENV mRNA expression from total RNA of MeT5A cells pre-treated with DMSO or MS-275 (0.25 μM) for 24 hours and were infected with SARS-CoV-2 for 72 hours (MOI=1). L34 mRNA levels were used for normalization. Four independent experiments were performed. P was calculated with respect to DMSO-treated MeT5A cells. Differences were considered significant at p < 0.05. **P< 0.01; ***P< 0.001; ns, not significant.

Since MS-275 induced an upregulation of SARS-CoV-2 receptors, we asked whether this could lead to an increased cell entry by the virus. Therefore, we used a VSV-spike-GFP pseudovirus (Case et al., 2020) and measure its entry in Met5A cells. As shown in **Figure 3A**, MS-275 treatment (0.25 μM) significantly enhanced viral entry.

We previously demonstrated the ability of MCs to support SARS-CoV2 infection and replication (Matusali et al., 2021). To analyze the role of HDAC1-3 inhibition in this process, MeT5A cells were treated with different concentrations of MS-275 or DMSO as a control for 24 hours before SARS-CoV-2 infection (MOI=1). As shown in **Figure 3B**, MS-275 treatment induced a significant increase of viral particles production in a dose-dependent manner. Accordingly, MS-275-treated cells (0.25 µM) have a higher expression of viral ENV RNA with respect to those treated with vehicle alone (**Figure 3C**).

These results demonstrated that HDAC1-3 pharmacological inhibition by MS-275 favors SARS-CoV-2, cell entry, replication and virion production in MeT5A cells.

### Treatment with MS-275 increases H3 and H4 histone acetylation on ACE2 and TMPRSS2 promoters

To mechanistically demonstrate an effect of HDAC1-3 inhibition in ACE2 and TMPRRS2 expression, the acetylation status of ACE2 and TMPRSS2 promoters was investigated.

**Figure 4:**
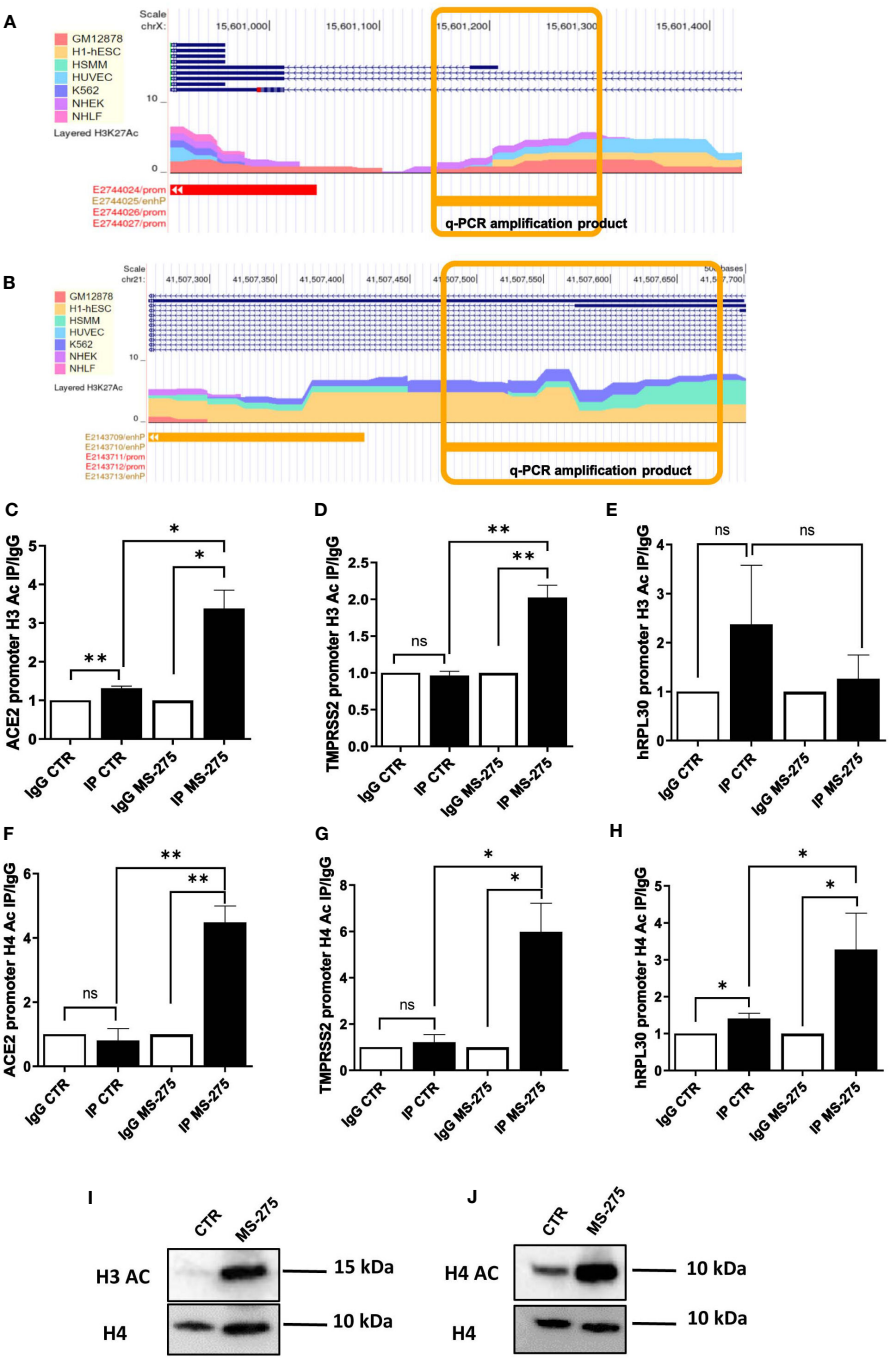
Treatment with MS-275 increases H3 histone acetylation on ACE2 and TMPRSS2 promoters. **(A, B)** Study of H3K27 acetylation of ACE **(A)** and TMPRSS2 promoter **(B)** from ENCODE (genome.ucsc.edu). Amplicons analyzed in panels C,D,F,G are underlined in yellow. **(C-E)** Magnetic ChIP experiment showing H3 acetylation on ACE **(C)**, TMPRSS2 **(D)** and hRLP30 promoter **(E)** upon treatment of MeT5A cells with MS-275 (0.25 μM) for 48 hours. qPCR of ChIP assays with anti- H3Ac and as control, with normal rabbit IgG on chromatin from MeT5A cells treated with DMSO (CTR) or with MS-275. **(F-H)** Magnetic ChIP experiment showing H4 acetylation on ACE **(F)**, TMPRSS2 **(G)** and hRLP30 promoter **(H)** upon treatment of MeT5A cells with MS-275 (0.25 μM) for 48 hours. qPCR of ChIP assays with anti- H4Ac and as control, with normal rabbit IgG on chromatin from MeT5A cells treated with DMSO (CTR) or with MS-275. Bars represent the mean ± SEM of triplicate determinations in at least three independent experiments. *P* was calculated with respect to IP CTR or IgG MS-275 samples. Differences were considered significant at *p* < 0.05. *P< 0.05; **P< 0.01; ns, not significant. **(I, J)** Western blot showing protein expression of acetylated H3 **(I)** and H4 **(J)** from histone extraction from MeT5A cells treated with MS-275. Total H4 was detected as a loading control. Images are representative experiment of three independent experiments.

The study of human ACE2 and TMPRSS2 enhancers/promoters from ENCODE revealed the presence of multiple acetylation peaks overlapping in 7 cell lines analyzed (**Figures 4A, B**). We then studied the acetylation status of histone H3 (Ac-H3) and H4 (Ac-H4) by specific chromatin immunoprecipitation (ChIP) assay.

Ac-H3 ChIP assay demonstrated that treatment with MS-275 at the concentration of 250 nM significantly enhances H3 acetylation on specific peak regions found on ACE2 and TMPRSS2 promoters (**Figures 4C, D**), but not on the promoter of hRLP30 (**Figure 4E**), a gene encoding a constitutively expressed ribosomal protein. Using the same experimental setting, an increase of H4 acetylation on specific sequences at ACE2 and TMPRSS2 promoters was found (**Figures 4F-H**).

Accordingly, MS-275 treatment promoted the acetylation on Histone H3 and H4, as shown by western blot analysis (**Figures 4I, J**). Overall, these results suggest that MS-275 promotes ACE2 and TMPRSS2 mRNAs expression by enhancing H3 and H4 acetylation, thus favoring an opened chromatin conformation in the promoter regions.

### Treatment with MS-275 increases ACE2/TMPRRS2 expression and potentiates SARS-CoV-2 replication and virion production in Calu-3 cells

**Figure 5:**
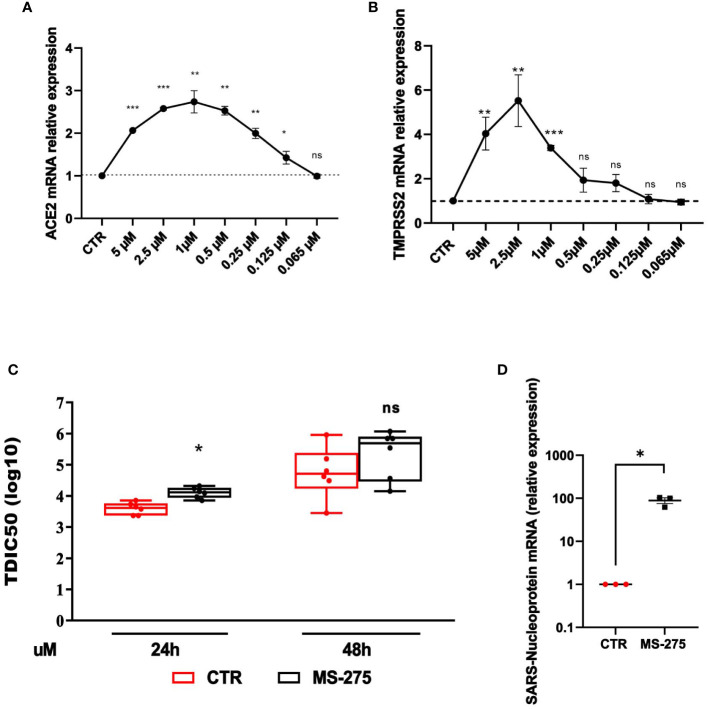
Treatment with MS-275 potentiates SARS-CoV-2 replication and productive infection in Calu-3 cells. **(A, B)** Calu-3 cells were treated with DMSO or MS-275 for 48 h at the following doses: 5, 2.5, 1, 0.5, 0.25, 0.125, 0.065 μM. Quantitative RT-PCR expression analysis of *ACE2* **(A)** and *TMPRSS2* **(B)** was performed on total RNA of MS-275-treated Calu-3 cells compared to DMSO-treated samples (CTR). L34 mRNA levels were used for normalization. Bars represent the mean ± SEM of triplicate determinations from three independent experiments. *P* was calculated with respect to CTR. Differences were considered significant at *p* < 0.05. Calu-3 cells were pre-treated with DMSO or MS-275 used at the concentrations shown in the image for 24 (h) Then, cells were infected with SARS-CoV-2 for 72 hours (MOI=0.001). **(C)** TCID50 (Median Tissue Culture Infectious Dose) assay was performed by adding serial dilutions of Calu-3 (MS-275- or DMSO-treated) cell culture supernatants to sub-confluent VeroE6 cells seeded in 96-well plates. Five independent experiments were performed. **(D)** Quantification of SARS-CoV-2 Nucleoprotein mRNA expression from total RNA of Calu-3 cells pre-treated with DMSO or MS-275 (1 μM) for 24 hours. Then, cells were infected with SARS-CoV-2 for 72 hours (MOI=1). L34 mRNA levels were used for normalization. Three independent experiments were performed. P was calculated with respect to DMSO-treated Calu-3 cells. Differences were considered significant at p < 0.05. *P< 0.05; **P< 0.01; ***P< 0.001; ns, not significant.

The analysis of the effect of HDAC1-3 inhibition was extended to Calu-3 cells, a lung adenocarcinoma cell line often used as an experimental system in SARS-CoV-2-related studies. Calu-3 cells express high levels of ACE2 and TMPRSS2 with respect to MeT5A cells (**Supplementary Figure 2**) (Liao et al., 2013; Najafi Fard et al., 2021; Alonzi et al., 2022). Similarly to the results obtained with MeT5A cells, treatment with MS-275 promoted a significant induction of both ACE2 and TMPRSS2 with a peak of induction at 1.0 µM and 2.5 µM, respectively (**Figures 5A, B**). The decreased levels of ACE2 and TMPRSS2 observed at higher doses of MS-275, are likely due to reduced cellular viability (**Supplementary Figure 1B**). According to the increased expression of receptors, pretreatment with MS-275 significantly increased both the TCID value, although at a less extent respect to that observed in MeT5A cells, and the expression of SARS-CoV-2 nucleoprotein (**Figure 5C, D**). These results demonstrate that besides cells from pleura, HDAC1-3 pharmacological inhibition by MS-275 favors SARS-CoV-2 replication and virion production also in human lung epithelial cells.

## Discussion

In this study, we describe a so far unrecognized effect of HDAC1-3 pharmacological inhibition in favoring SARS-CoV-2 replication and virion production in MCs. These results correlated with increased ACE2 and TMPRSS2 gene expression and with enhanced H3 and H4 acetylation on their promoters.

Epigenetics may modulate different aspects of SARS-CoV-2 pathogenesis, including factors linked with virus-cell interaction, cell spreading, and development of an inflammatory/immune response. We focused on the regulation of ACE2 and TMPRSS2 expression since ACE2 is the first determinant of SARS-COV-2 entry in respiratory airways. In macrophages, ACE2 expression is necessary for SARS-COV-2 infection and subsequent secretion of inflammatory cytokines (Labzin et al., 2023). Indeed, ACE2 expression in the lungs correlates with severe COVID-19 (Pinto et al., 2020), and human recombinant soluble ACE2 (hrsACE2) may block SARS-CoV-2 infection (Monteil et al., 2020; Monteil et al., 2022).

The modulation of ACE2 expression by HDAC inhibitors has been analyzed in previous studies. Treatment with Sodium butyrate and Panobinostat downregulated ACE2 expression in a gastric cancer cell line (Takahashi et al., 2021). Valproic acid (VPA) reduced ACE2 expression in different epithelial and endothelial cell lines (Saiz et al., 2021), and TMPRSS2 expression in prostate cancer cells (Fortson et al., 2011).

Differently from most studies analyzed, we found that almost all the pharmacological inhibitors tested in our experimental system increased ACE2 and TMPRSS2 expression, although the effect was strongly variable between the different selective HDAC inhibitors.

The best scores were obtained with MS-275 and MGCD0103, an HDAC1-3 and HDAC1/2 selective inhibitor, respectively (Saito et al., 1999; Fournel et al., 2008; Khan et al., 2008). The role of HDAC1 and HDAC2 in the modulation of ACE2 and TMPRSS2 expression was further validated by specific genetic silencing. The fact that the HDAC3-selective inhibitor MC4448 induced the expression of both ACE2 and TMPRSS2, this may highlight the additive role of HDAC3 inhibition in producing this effect.

Of note, MC2500, the inactive *meta* isomer of MS-275, was ineffective (negative control). The role of HDAC1 and HDAC2 in the modulation of ACE2 and TMPRSS2 expression was further validated by specific genetic silencing.

Although several studies dealt with the impact of HDAC inhibitors in the regulation of ACE2 and TMRPRSS2 expression, there is a lack of mechanistic details analyzing their impact on the histone acetylation at ACE2 and TMRPRSS2 promoters. HDAC2 and other epigenetic modifiers were predicted as potential regulators of ACE2 from correlation and network analyses of lung transcriptome from COVID-19 patients (Pinto et al., 2020). In another study, the NAD^+^-dependent HDAC SIRT1 was found to interact with the ACE2 promoter favoring ACE2 expression under conditions of cell energy stress (Clarke et al., 2014).

Thus, we analyzed in detail the acetylation status of the ACE2 promoter. We found that MS-275 treatment promoted a significant increase in histone H3 and H4 acetylation at both ACE2 and TMPRSS2 promoters. It is well known that histone H3 and H4 acetylation promotes the acquisition of an open conformation of DNA, generally increasing the transcriptional activity of the adjacent DNA.

Moreover, we extended the results obtained in MeT5A to a human lung epithelial cellular line, Calu-3 cells, widely used in SARS-CoV-2 studies. Similarly to MeT5A cells, we found an increase in ACE2 and TMRSS2 expression upon treatment with MS275 which correlated with increased viral replication at early time points. However, we noticed that albeit significant, the increase in receptor expression and in TCID values were in Calu-3 cells not as strong as in MeT5A cells. Since ACE2 and TMPRSS2 expression is more abundant at basal condition (unstimulated condition) in Calu-3 than in MeT5A cells, we speculated that treatment with MS-275 acting on ACE2/TMPRSS2 expression may favor virus infection and replication in cells expressing at basal conditions low levels of receptors, thus favoring viral spreading to secondary sites of infection.

Besides HDACs, ACE2 expression has been demonstrated to be regulated by other epigenetic modulators (Rath et al., 2021; Sen et al., 2021). DNA methylation has been correlated to ACE2 expression. *ACE2* gene expression was regulated by changes in the DNA methylation patterns in its promoter region (Daniel et al., 2022). In another study, the DNA methylation profile of ACE2 and TMPRSS2 promoter regions was analyzed (Subbarayan et al., 2021).

Histone methylation was also assessed: loss of function of the histone methyltransferase regulating the H3K27me3 repressive mark correlates to enhanced ACE2 expression in mouse germline cells (Li Y. et al., 2020). Moreover, Lysine-specific demethylase 1 (LSD1) was shown to couple to ACE2 at the plasma membrane following SARS-CoV-2 infection, being critical for the regulation of the ACE2–SARS-CoV-2 interaction required for viral entry (Tu et al., 2021).

Besides regulating ACE2 expression, HDACs may impact SARS-CoV-2 infectivity and viral replication in different ways (Dey et al., 2023). Several HDAC inhibitors were found to control SARS-CoV-2-S pseudovirus cell entry via inhibition of clathrin-mediated endocytosis (Liu et al., 2020). HDAC inhibition may modulate inflammatory cytokine production in the epithelial cells of the respiratory tract (Ripamonti et al., 2022). HDAC6-selective inhibition has been proposed as a therapeutic strategy to restore the immune response in severe COVID-19 patients (Ripamonti et al., 2022).

The activity of class I HDAC inhibitors has also been linked to immunomodulatory effects. MS-275 has been demonstrated to promote activation of intra-tumoral CD8 T cells (Hicks et al., 2021; McCaw et al., 2021). We and others previously demonstrated an antifibrotic role of MS-275 and other class I inhibitors in MCs and other cellular types, which is potentially beneficial in the therapy of lung fibrosis, a frequently reported COVID-19 sequela (Kim et al., 2018; Rossi et al., 2018; McGroder et al., 2021; Bontempi et al., 2022).

MS-275 is currently tested in combination therapy in different clinical trials for both solid tumors and lymphomas (Connolly et al., 2017; Feng and De Carvalho, 2022). However, treatments for COVID-19 or other viral diseases may interfere or cross-react with other ongoing treatments, especially in immunocompromised patients: concerns about the safety of using anticancer drugs during COVID-19 have been raised (Gougis et al., 2020).

We here demonstrate that treatment with selective HDAC1-3 inhibitors increases the expression of ACE2 and TMPRSS2, likely favoring viral spreading, especially to cells with low expression of these receptors, and in turn, promoting the colonization of new tissues and the chronicization of the infection.

Our results raise a concern about the use of MS-275 or related HDAC1-3 inhibitors in patients, where it may further potentiate the spread of SARS-CoV-2 infection.

## Data availability statement

The original contributions presented in the study are included in the article/**Supplementary Material**. Further inquiries can be directed to the corresponding authors.

## Ethics statement

Ethical approval was not required for the studies on humans in accordance with the local legislation and institutional requirements because only commercially available established cell lines were used. Ethical approval was not required for the studies on animals in accordance with the local legislation and institutional requirements because only commercially available established cell lines were used.

## Author contributions

RS: Funding acquisition, Supervision, Writing – original draft, Writing – review & editing, Project administration. FT: Conceptualization, Formal analysis, Investigation, Writing – review & editing. TA: Conceptualization, Formal analysis, Investigation, Writing – review & editing. MTe: Conceptualization, Investigation, Writing – original draft. GB: Investigation, Writing – original draft. CB: Methodology, Supervision, Validation, Writing – review & editing. CM: Methodology, Software, Writing – original draft. FR: Investigation, Methodology, Writing – original draft. DR: Formal analysis, Resources, Supervision, Writing – review & editing. SV: Formal analysis, Funding acquisition, Resources, Supervision, Writing – review & editing. CZ: Formal analysis, Software, Writing – review & editing. GM: Conceptualization, Investigation, Methodology, Writing – review & editing. FM: Funding acquisition, Supervision, Writing – review & editing. DG: Funding acquisition, Supervision, Writing – review & editing. MTr: Conceptualization, Supervision, Writing – review & editing. AM: Conceptualization, Supervision, Writing – review & editing.
