# Supplementary figures and images for: HDAC1-3 inhibition increases SARS-CoV-2 replication and productive infection in lung mesothelial and epithelial cells
Source: Front Cell Infect Microbiol. 2023 Dec 13;13:1257683. doi: 10.3389/fcimb.2023.1257683 (PMC10757821; doi:10.3389/fcimb.2023.1257683)

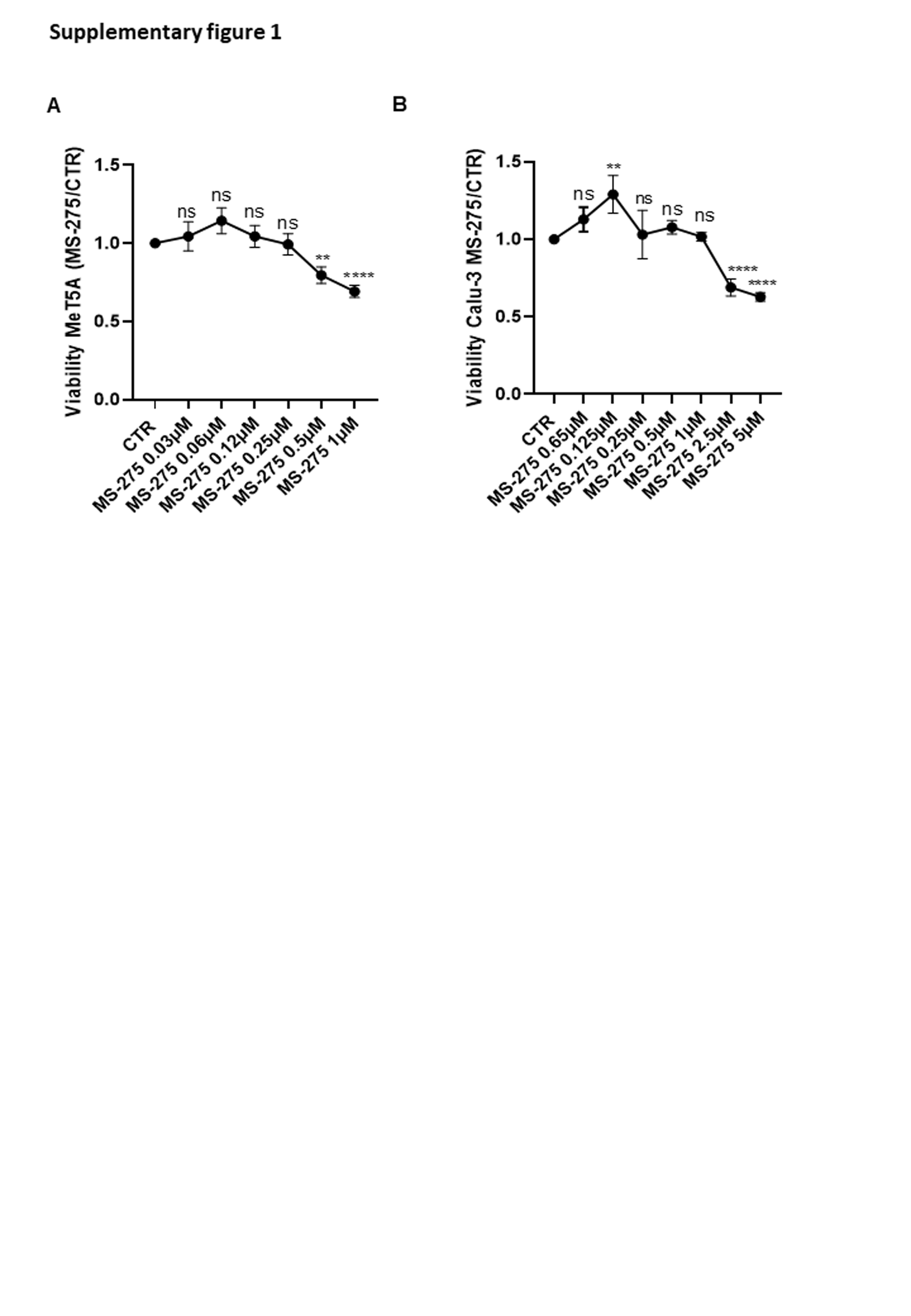

Supplement: Supplementary file 1 [file Image_1.tif]

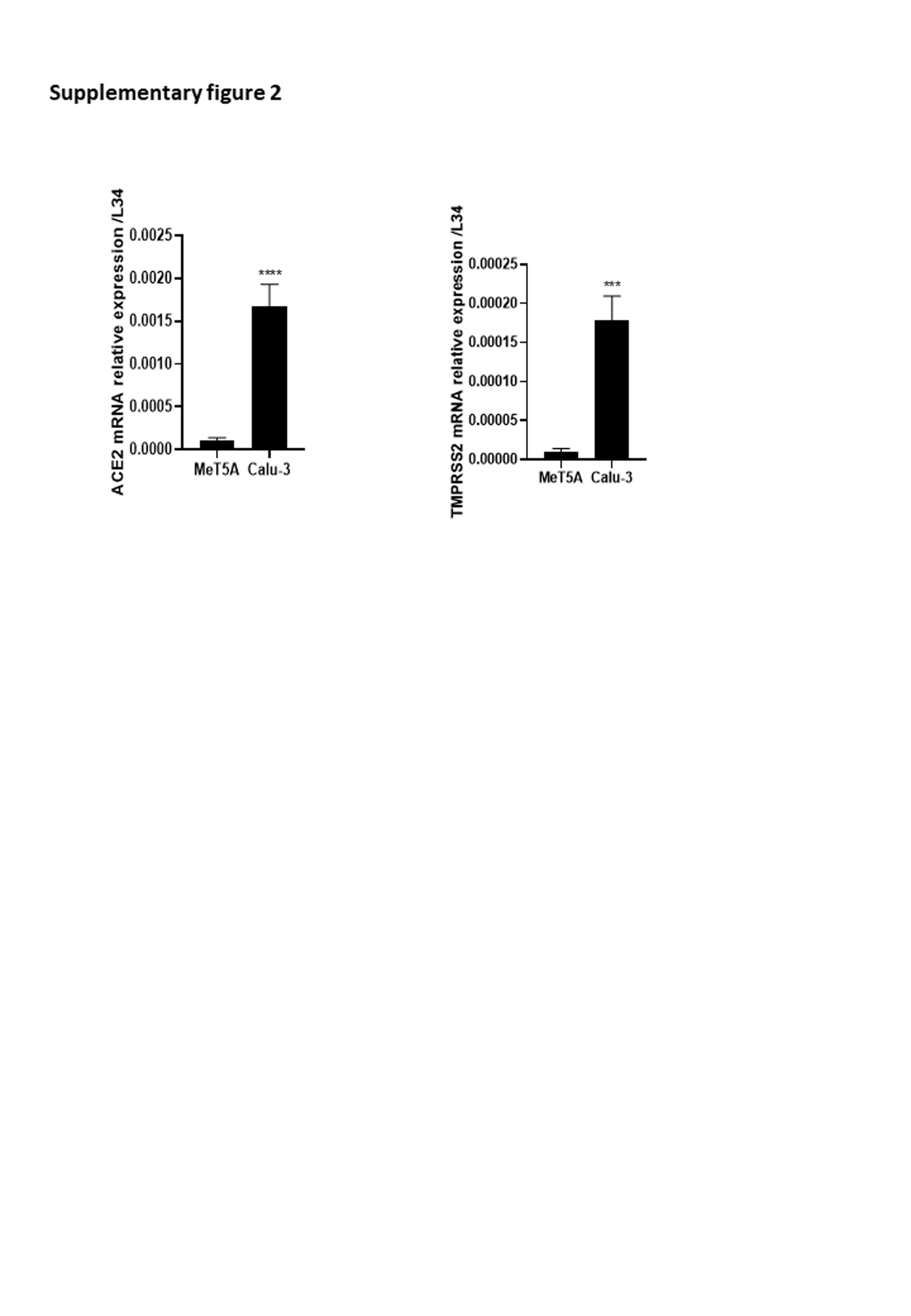

Supplement: Supplementary file 2 [file Image_2.tif]
